# Age-related upregulation of p16 expression in mouse ovarian somatic cells correlated with reproductive function decline p16 expression and ovarian aging in mice

**DOI:** 10.1371/journal.pone.0348870

**Published:** 2026-05-08

**Authors:** Yorino Sato, Yuta Kawagoe, Kazuhiro Kawamura

**Affiliations:** Department of Obstetrics and Gynecology, Juntendo University Faculty of Medicine, Tokyo, Japan; University of Newcastle, UNITED KINGDOM OF GREAT BRITAIN AND NORTHERN IRELAND

## Abstract

Female reproductive aging is a major clinical challenge associated with declining fertility and increased pregnancy complications. The urgent clinical need for developing reliable biomarkers to evaluate ovarian aging has become increasingly evident. Cellular senescence, marked by p16, contributes to age-related tissue dysfunction. However, the relationship between p16 levels and ovarian aging remains poorly understood. Age-related changes in p16 levels across multiple tissues in ICR mice were examined in ICR mice at 4, 30, 45, and 60 weeks of age using qRT-PCR, ELISA, and immunohistochemistry. Cell-type specific p16 levels were analyzed in isolated ovarian cells. Reproductive function was assessed through superovulation, in vitro fertilization, and embryo transfer experiments. p16 mRNA levels increased progressively with age in ovarian tissue (6.8-fold increase at 60 weeks vs. 4 weeks, P < 0.05), with corresponding increases in p16 protein levels. Among tissues examined, ovaries, kidneys, liver, uterus, spleen, and pancreas showed significant age-related p16 upregulation, while brain, heart, and lung did not. Cell-type analysis revealed that somatic cells exhibited pronounced p16 upregulation with age (cumulus cells: 3.2-fold, granulosa cells: 4.6-fold, theca cells: 2.8-fold increase), whereas oocytes and blastocysts showed no significant changes. Ovulation numbers decreased significantly with age (42.3 ± 3.1 vs. 15.6 ± 1.9 oocytes in young vs. aging mice), but fertilization rates and early embryo development remained unaffected. However, post-implantation outcomes deteriorated substantially, with implantation rates declining from 78.4% to 38.1% and live birth rates from 82.3% to 43.2% in aging mice at 60 weeks of age. Age-related upregulation of p16 in ovarian somatic cells, but not in oocytes, correlated with declining reproductive function, particularly affecting post-implantation development. These findings suggest that somatic cell senescence may contribute to age-related declines in oocyte competence, leading to fertility decline with aging.

## Introduction

Ovarian aging refers to the age-related decline in ovarian function, characterized by a progressive decrease in oocyte quality and a reduction in female reproductive competence. The ovaries exhibit functional aging relatively early in a woman#39;s lifetime, significantly limiting reproductive capacity [1,2]. However, clinically established methods for objectively evaluating oocyte quality remain limited in current medical practice, and non-invasive and accurate assessment of oocyte and somatic cell aging remains a particularly challenging task. The development of reliable biomarkers for evaluating the aging status of oocytes and ovarian somatic cells is urgently needed for advances in female reproductive medicine.

A potential biomarker for ovarian aging is p16INK4a (p16), encoded by the *Cdkn2a* gene, which halts cell cycle progression from G1 to S phase by inhibiting cyclin-dependent kinases CDK4 and CDK6 [1,2]. p16 is widely recognized as a robust biomarker of cellular senescence, as its levels increase dramatically when cells reach their replicative limit or encounter stress-induced growth arrest [3]. Krishnamurthy et al. demonstrated that *Ink4a/Arf* expression markedly increases with advancing age across multiple rodent tissues—including the kidney, liver, and notably the ovary—while other CDK inhibitors showed little age-related change [3]. Importantly, the age-associated increase in p16 levels in the kidney, ovary, and heart was attenuated by caloric restriction, with a corresponding reduction in senescence-associated pathology [3]. These findings established p16 as a biomarker and possible effector of mammalian aging, indicating that elevated p16 levels accumulate as a consequence of aging-related cellular stress rather than serving a homeostatic maintenance function in young tissues [3,4].

In the human ovary, p16 protein levels have been predominantly reported in relation to ovarian cancer, where this important tumor suppressor is frequently mutated or shows loss of function [5,6]. However, the role of p16 in physiological ovarian aging has received increasing attention. Research on p16 in the context of ovarian biology has spanned over two decades. Bayrak and Oktay reported that p16 protein levels varied depending on the follicular stage in the mouse ovary: p16 staining was strong in oocytes of primordial follicles and in approximately half of primary follicles, becoming weaker in more advanced follicles [7]. Strong p16 staining was also observed in the oocytes of atretic follicles, suggesting a potential role in cell cycle arrest contributing to follicular atresia [7]. Subsequent studies have further linked p16 accumulation to ovarian dysfunction. Nie et al. demonstrated that consecutive superovulation in mice induced premature ovarian failure accompanied by significantly elevated p16 protein levels, along with increased oxidative stress and granulosa cell apoptosis via the SIRT1/FOXO1 pathway [8]. Ansere et al. showed that cellular senescence markers, including *Cdkn2a* transcripts, accumulate in mouse ovaries with advancing age, preceding the dramatic decline in primordial follicle reserve [9]. More recently, Huang et al. reported that long-term nicotinamide mononucleotide treatment improved age-related diminished ovarian reserve through enhanced mitophagy in granulosa cells, suggesting that senescence-related pathways may represent therapeutic targets for ovarian aging [10]. Furthermore, Secomandi et al. provided a comprehensive review of cellular senescence in female reproductive aging, exploring senolytic and senostatic therapies for extending reproductive longevity [11]. A recent single-nuclei multi-omics study of human ovarian tissue revealed that *CDKN1A* (p21)-positive cells increase with age across multiple cell types, while *CDKN2A* (p16)-expressing cells were detected at lower frequency [12]. Additionally, Watson et al. mapped p16-positive senescent cells in postmenopausal human ovaries using spatial transcriptomics and identified distinct stromal clusters associated with fibrosis and inflammatory gene signatures [13]. Despite these advances, the systematic characterization of cell-type-specific changes in p16 levels across the full reproductive lifespan—distinguishing between ovarian somatic cells and oocytes—and their direct correlation with functional reproductive outcomes has not yet been fully elucidated.

**Fig 1:**
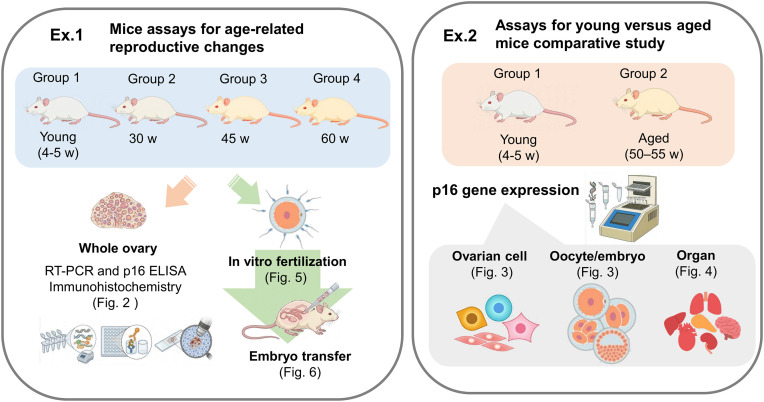
Schematic overview of the experimental design. Schematic overview of the experimental design. This study comprised two study arms. Left: Mice assays for age-related reproductive changes. Four groups of female ICR mice (young [4–5 weeks], 30 weeks, 45 weeks, and 60 weeks) were used for whole ovarian tissue collection for qRT-PCR and p16 ELISA (n = 6–8 animals/group) and immunohistochemistry (n = 3 animals/group) (Fig 2), in vitro fertilization using female mice from groups 1–4 (n = 10–15 animals/group) with male sperm donors (n = 4 animals) (Fig 5), and embryo transfer to pseudopregnant foster mothers (n = 10–15 animals/group) (Fig 6). Right: Assays for young versus aged mice comparative study. Two groups (young [4–5 weeks] and aged [50–55 weeks]) were used for ovarian cell specific p16 levels (ovarian cells obtained from 8 animals, 6–8 samples/group) (Fig 3), oocyte/embryo specific p16 levels (n = 5 oocytes or embryos/sample, 6–8 samples/group) (Fig 3), and organ specific p16 levels (n = 8 animals/group) (Fig 4).

This study aimed to elucidate the age-associated changes in p16 levels within ovarian tissue and to clarify the correlation between elevated p16 levels in specific ovarian cell types and age-related decline in reproductive function. Specifically, we investigated *Cdkn2a* gene expression and p16 protein levels across multiple age groups in ICR mice, with a particular focus on distinguishing the contributions of ovarian somatic cells (granulosa cells, cumulus cells, and theca cells) from those of oocytes. Through this cell-type-specific approach, combined with functional assessment by *in vitro* fertilization and embryo transfer, we sought to evaluate the potential utility of p16 as a biomarker for age-related ovarian dysfunction (Fig 1).

## Materials and methods

### Animals

Female ICR mice purchased from Sankyo Labo Service Corporation, Inc. (Tokyo, Japan) were divided into four age groups: young (4–5 weeks of age) and aged (30, 45, and 60 weeks of age). The five male mice for in vitro fertilization were ICR mice aged 10–12 weeks. All animals were housed under standard laboratory conditions with controlled temperature (22 ± 2°C), humidity (55 ± 5%), and a 12-hour light/dark cycle. Animals had free access to standard rodent chow and water. The animal experiments were conducted following the guidelines of Juntendo University and were approved by the Institutional Animal Care and Use Committee of Juntendo University (Permit No. 2022288). All mice were euthanized by cervical dislocation performed by a trained individual to ensure humane treatment. Throughout the study, all procedures were conducted in accordance with the ARRIVE guidelines to minimize animal distress, and humane endpoints were established.

## Experimental design

### Experiment 1: Age-specific comparative study

The first experiment aimed to characterize age-related changes in p16 levels across ovarian tissue and to assess the impact of aging on reproductive function. Female ICR mice were divided into four age groups: young (4–5 weeks), 30 weeks, 45 weeks, and 60 weeks. For molecular analyses, 10 mice per age group were euthanized, and ovaries and other organs (kidneys, spleen, liver, uterus, brain, lungs, pancreas, and heart) were collected. Ovaries for RNA extraction and ELISA were snap-frozen in liquid nitrogen and stored at −80°C (n = 6–8 per group for each assay). Ovaries for immunohistochemistry were fixed in Bouin#39;s solution (n = 3 per group). For functional assessment of reproductive capacity, a separate cohort of 10–15 mice per age group underwent ovulation induction, oocyte collection, in vitro fertilization, and embryo transfer. To avoid confounding from differences in embryo numbers, 7.43 ± 0.88 blastocysts were transferred per surrogate mother.

### Experiment 2: Young versus aged comparison

The second experiment aimed to identify cell-type-specific changes in p16 levels between young and aged ovaries and to compare organ-specific p16 accumulation. Two age groups were used: young (4–5 weeks) and aged (50–55 weeks). For cell-type-specific analysis, ovaries from 8 mice per age group were collected, and ovarian somatic cells (cumulus cells, granulosa cells, theca cells, corpus luteum, and corpus albicans) were mechanically isolated for qRT-PCR analysis (n = 6–8 per group). To analyze p16 levels in oocytes and embryos, MII oocytes and blastocysts were collected via IVF using three female donors and one male donor per age group (n = 6–8 per group). For organ-specific comparison, tissues from 8 mice per age group were collected and snap-frozen for qRT-PCR. Where possible, the same animals were used for multiple analyses to minimize animal numbers in accordance with the 3Rs principle.

### RNA Extraction and Quantitative Real-Time PCR

Total RNA was extracted from various tissues (ovaries, kidneys, spleen, liver, uterus, brain, lungs, pancreas, heart) using the Qiagen RNeasy Kit (Qiagen, Hilden, Germany). For reproductive cell-specific analysis, mechanical separation using a 27G needle-tipped syringe as previously described [14,15] was performed to isolate cumulus cells (CC), granulosa cells (GC), theca cells (TC), corpus luteum (CL), and corpus albicans (CA). Cell purity was confirmed by RT-PCR analysis using cell-specific markers for *Gdf9* for oocytes, *Has2* for cumulus cells, *Fsh* receptor for granulosa cells, and *Cyp17a1* for theca cells. Mature oocytes (MII) were collected 14–16 hours after administration of hCG 15 IU (Aska Animal Health Co., Ltd., Tokyo, Japan), and blastocysts (BL) were harvested 96 hours after in vitro fertilization.

Total RNA was reverse transcribed into cDNA using the PrimeScript RT Reagent Kit (Takara Bio, Shiga, Japan). Quantitative PCR was performed using SYBR Green PCR master mix (Roche, Basel, Switzerland) on a real-time PCR system (Roche, Basel, Switzerland). The relative levels of p16 were normalized using *Gapdh* as an internal control via the 2^(-ΔΔCt) method. The primer sequences for p16 and *Gapdh* were as follows: p16: Forward 5’-CGTACCCCGATTCAGGTGAT-3,’ Reverse 5’-TTGAGCAGAAGAGCTGCTACGT-3,’ *Gapdh*: Forward 5’-GTGGCAAAGTGGAGATGGTTGCC-3,’ Reverse 5’-GATGATGACCCGTTTGGCTCC-3’

### Tissue processing and protein extraction

Ovarian tissue was collected from female mice of different age groups (3, 30, 45, and 60 weeks of age) and rapidly frozen in liquid nitrogen, then stored at −80°C. Following the manufacturer#39;s instructions, 30 mg of tissue was homogenized in 300 μL of ice-cold cell extraction buffer, and 50 μL of each tissue lysate was applied per well. Measurement of total protein concentration was omitted in accordance with the kit protocol (Abcam, ab230131). The homogenate was incubated on ice for 30 minutes, then centrifuged at 4°C at 14,000 × g for 20 minutes. The protein-containing supernatant was collected, aliquoted, and stored at −80°C.

### p16 ELISA

Mouse p16 protein levels were quantified using the Mouse *CDKN2A*/p16INK4a ELISA Kit (Abcam, Cambridge, UK). For ELISA, 50 μl of each lysate was loaded per well and p16 concentrations were calculated against the kit’s standard curve (22–1400 pg/ml), as instructed by the manufacturer. Standards and samples were added to pre-coated wells and incubated at room temperature for 1 hour with gentle shaking. HRP-streptavidin solution was added and incubated for 45 minutes. TMB substrate reagent was then added, and the color development reaction was performed for 10 minutes in the dark. The reaction was stopped with stop solution, and absorbance was measured at 450 nm. The concentration of p16 was calculated using a standard curve (range: 22 pg/ml – 1400 pg/ml). All analyses were performed in duplicate.

### Evaluation of oocyte quality by in vitro fertilization and embryo transfer

Mice were administered 15 IU of human chorionic gonadotropin (hCG; Asuka Animal Health Co., Ltd.) via intraperitoneal injection to induce ovulation, with no PMSG used for either group. After hCG administration, cumulus-oocyte complexes (COCs) were collected from the oviducts 14–15 hours later and co-cultured with TSH-containing fertilization medium (PHC Holdings Corporation, Tokyo, Japan) with sperm capable of fertilization (2–3 × 10⁵ sperm/ml) and cultured for 6 hours at 37°C and 5% CO₂. Fertilization-competent sperm were collected from the cauda epididymis, and motile sperm were selected by the swim-up method. The fertilization rate was assessed by counting the number of two-cell embryos at 24 hours after insemination. Fertilized oocytes were cultured in KSOM medium (Millipore, Burlington, MA) under mineral oil (Sigma-Aldrich, St. Louis, MO) at 37°C in 5% CO₂ from the 2-cell stage (24 h after insemination) until the blastocyst stage (96 h after insemination). Embryo development was monitored daily, and the developmental rates at the 4-cell stage, 8-cell stage, morula stage, and blastocyst stage were calculated based on the proportion of 2-cell embryos.

Embryo transfer was performed by transferring blastocyst-stage embryos into the uterus of purchased pseudopregnant surrogate mothers. Mating success was confirmed by the presence or absence of a vaginal plug. Female mice were euthanized on day 18 of pregnancy, and the number of implantation sites, litter size, pup weight, and placental weight were measured. The implantation rate was calculated as the number of implantation sites divided by the number of transferred embryos. The survival rate was calculated as the number of live fetuses divided by the number of implantation sites, and the miscarriage rate as the number of resorbed or dead fetuses divided by the number of implantation sites.

### Immunohistochemistry

The ovaries used for IHC were collected from mice at each defined age (4–5, 30, 45, 60 weeks of age). And ovaries were fixed in Bouin#39;s solution (FUJIFILM Wako Pure Chemical Corporation, Osaka, Japan) for 24 hours, embedded in paraffin, and sectioned at 6 μm thickness using a rotary microtome (YAMATO KOHKI INDUSTRIAL Inc., Saitama, Japan). Sections were deparaffinized with xylene (Nacalai Tesque, Kyoto, Japan) and rehydrated through a graded ethanol series (Nacalai Tesque) (100%, 95%, 80%, 70%). Antigen retrieval was performed by heating sections at 95°C for 20 minutes in 10 mM citrate buffer (pH 6.0). Endogenous peroxidase activity was quenched by treating sections with 3% H₂O₂ in methanol (Sigma-Aldrich, St. Louis, MO, USA) for 10 minutes at room temperature. Non-specific binding was blocked by incubating sections with blocking solution (Cell Signaling Technology, Danvers, MA, USA) for 1 hour at room temperature. Sections were incubated with anti-p16 primary antibody (1:200 dilution; Abcam, Cambridge, UK) overnight at 4°C in a humid chamber. After washing with PBS (3 × 5 minutes), sections were incubated with HRP-conjugated anti-rabbit secondary antibody (1:500 dilution; Abcam) for 1 hour at room temperature. Immunoreactivity was visualized using the HRP/AEC Detection IHC Kit (Abcam). All images were captured using an all-in-one fluorescence microscope (Keyence, Osaka, Japan) in bright-field mode after AEC chromogenic immunohistochemistry. Immunohistochemical analysis was performed for qualitative assessment of p16 protein distribution across ovarian tissue compartments. Representative images were selected to illustrate age-related changes in p16 staining patterns.

### Statistical analysis

Data are presented as mean ± standard error of the mean (SEM). For comparisons between two groups, unpaired Student#39;s t-test was used. For multi-group comparisons (e.g., age-dependent changes), one-way analysis of variance (ANOVA) was performed, followed by Tukey#39;s post-hoc test to determine significant differences between individual groups. Statistical analyses were performed using GraphPad Prism 9.0 software (GraphPad Software Inc., San Diego, CA, USA). A value of P < 0.05 was considered statistically significant. The specific statistical test used for each experiment is indicated in the corresponding figure legend.

## Results

### Age-dependent upregulation of ovarian p16 levels in mice

**Fig 2:**
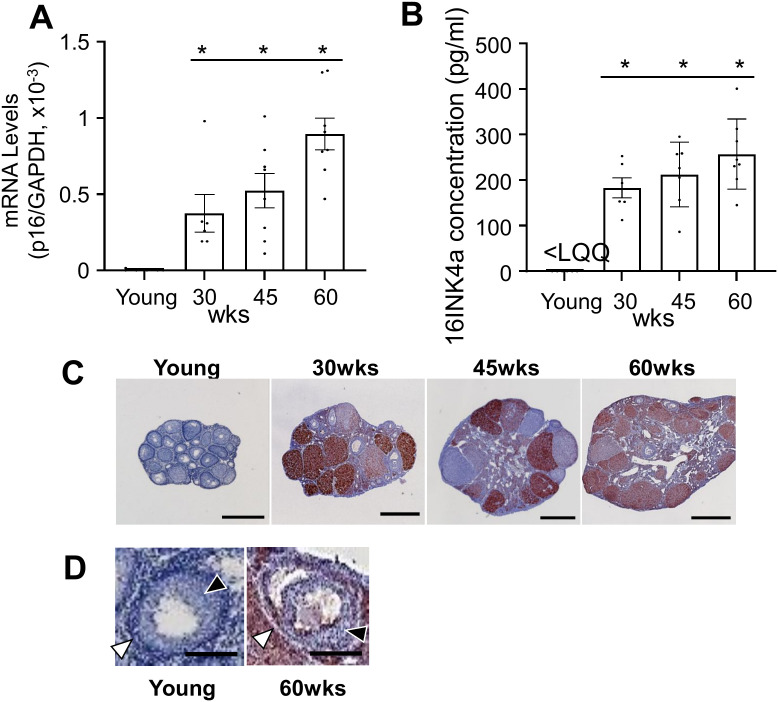
Age-dependent increase in p16 levels in mouse ovary. Gene expression analysis of p16 in mouse ovaries by quantitative real-time PCR (qPCR). p16 mRNA levels in the ovaries of young (4–5 weeks of age), 30-, 45-, and 60-weeks of age mice were normalized for GAPDH (n = 6–8 per group). Data were analyzed by one-way ANOVA followed by Tukey#39;s post-hoc test (*p < 0.05). (B) p16INK4a protein concentration (pg/ml) measured by ELISA (n = 6–8 per group; < LQQ, below the limit of quantification). Data were analyzed by one-way ANOVA followed by Tukey#39;s post-hoc test (*p < 0.05). (C) Representative immunohistochemical staining for p16 in ovarian sections of young, 30-, 45- and 60- week of age mice (n = 3 per group, scale bar = 1 mm). (D) Representative higher-magnification images of follicles showing p16 staining in young and 60-week-old follicles (scale bar = 100 μm). Black arrowheads indicate granulosa cells (GC); white arrowheads indicate theca cells (TC). wks: weeks.

**Fig 3:**
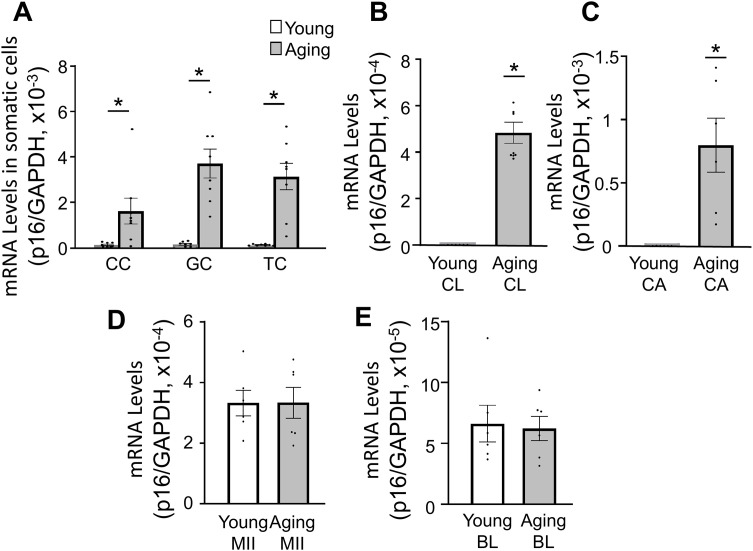
Cell-type specific p16 level changes with aging. mRNA levels increased with age in somatic cells but did not change in oocytes. Changes in mRNA levels in ovarian component cells in young (4–5 weeks of age) and aging (50–55 weeks of age) mice. (A) Cumulus cell (CC), granulosa cell (GC) and theca cell (TC). (B) Corpus luteum (CL). (C) corpus albicans (CA). (D) MII oocyte. (E) blastocyst-stage embryo (BL). For all samples, n = 6–8 per group. Values are normalised by GAPDH. Data are presented as mean ± SEM. Statistical analysis was performed using Student#39;s t-test (*p < 0.05 compared to young control).

To evaluate the impact of aging on cellular senescence in the ovary, p16 levels in mouse ovaries were assessed. Quantitative RT-PCR analysis demonstrated a significant, progressive increase in p16 mRNA levels with advancing age (Fig 2A; P < 0.05 for all comparisons with young controls). Consistent with the mRNA data, ELISA measurements revealed a corresponding age-dependent elevation in p16 protein levels (Fig 2B; P < 0.05). Detectable p16 protein was present in 78% (9/12) of 30 and 45 weeks of age and 95% (10/11) of 60 weeks of age ovarian samples, whereas the majority of young samples (85%, 7/8) were below the quantification limit (< 5 pg/ml). Immunohistochemical analysis further corroborated these findings, demonstrating a progressive increase in p16 staining intensity in ovarian sections from aging mice compared to young controls (Fig 2C). Furthermore, consistent with the cell type-specific analysis shown in Fig 3, the age-related increase in p16 staining depicted in Fig 2C was primarily observed in somatic regions, including the granulosa layer and theca layer of the follicle (Fig 2D).

### Differential age-related p16 levels in ovarian somatic cells and germ cells

To identify the ovarian somatic cell populations responsible for age-related p16 upregulation, p16 levels in isolated cell types were analyzed from young (4–5 weeks of age) and aging (50–55 weeks of age) mice. In all somatic cell types examined, p16 transcript levels were elevated with age (Fig 3A-C). Among the follicular somatic cells, granulosa cells exhibited the greatest age-associated increase in p16 levels with a 4.6-fold higher levels in aging compared to young controls. Cumulus cells and theca cells showed 3.2-fold and 2.8-fold higher levels, respectively, in aging mice relative to young mice (Fig 3A). In post-ovulatory ovarian samples, both corpus luteum (5.1-fold higher levels in aging, P < 0.05) and corpus albicans (7.3-fold higher levels in aging, P < 0.05) demonstrated substantial age-dependent upregulation of p16 (Fig 3B and C).

In contrast, p16 mRNA levels in metaphase II (MII) oocytes did not differ between young and aging groups (Fig 3D). Similarly, blastocyst-stage embryos derived from oocytes of aging animals showed no significant age-related changes in p16 levels (Fig 3E).

### Organ-specific patterns of age-related p16 levels

**Fig 4:**
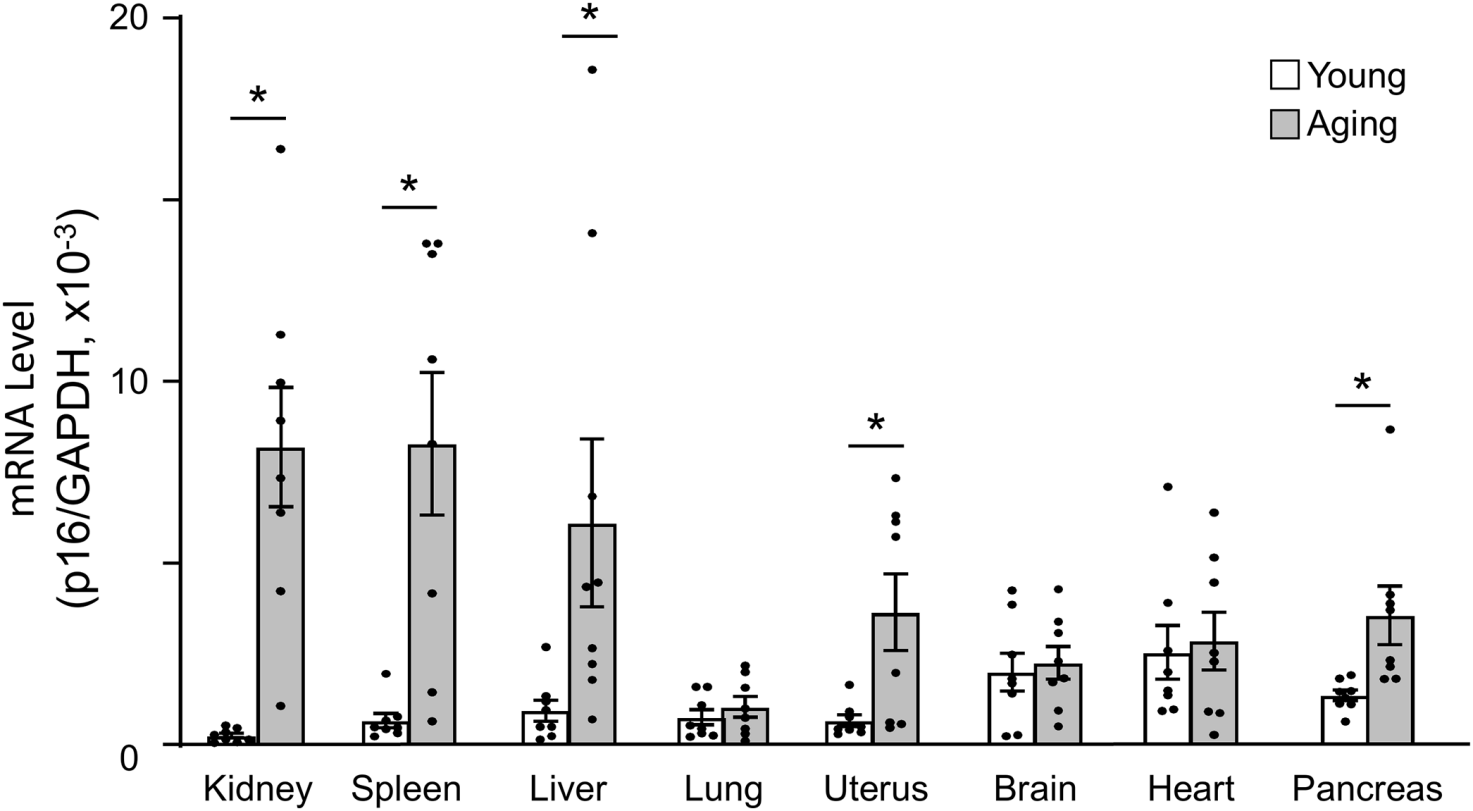
Tissue-specific patterns of age-related p16 levels. Comparison of p16 mRNA levels in different tissues in young (4–5 weeks of age) and aging (50–55 weeks of age) mice. mRNA levels of p16 normalised by GAPDH (n = 8 per group). Data are presented as mean ± SEM and analyzed using Student#39;s t-test (*p < 0.05, young vs aging).

The analysis revealed distinct, organ-specific patterns of age-related changes in p16 levels (Fig 4). Among the examined tissues, a marked increase in p16 levels with ageing was observed in the ovaries, kidneys, spleen, liver, uterus, and pancreas, but not in the brain, lungs, or heart.

### Age-related declines in ovarian function and reproductive outcomes

**Fig 5:**
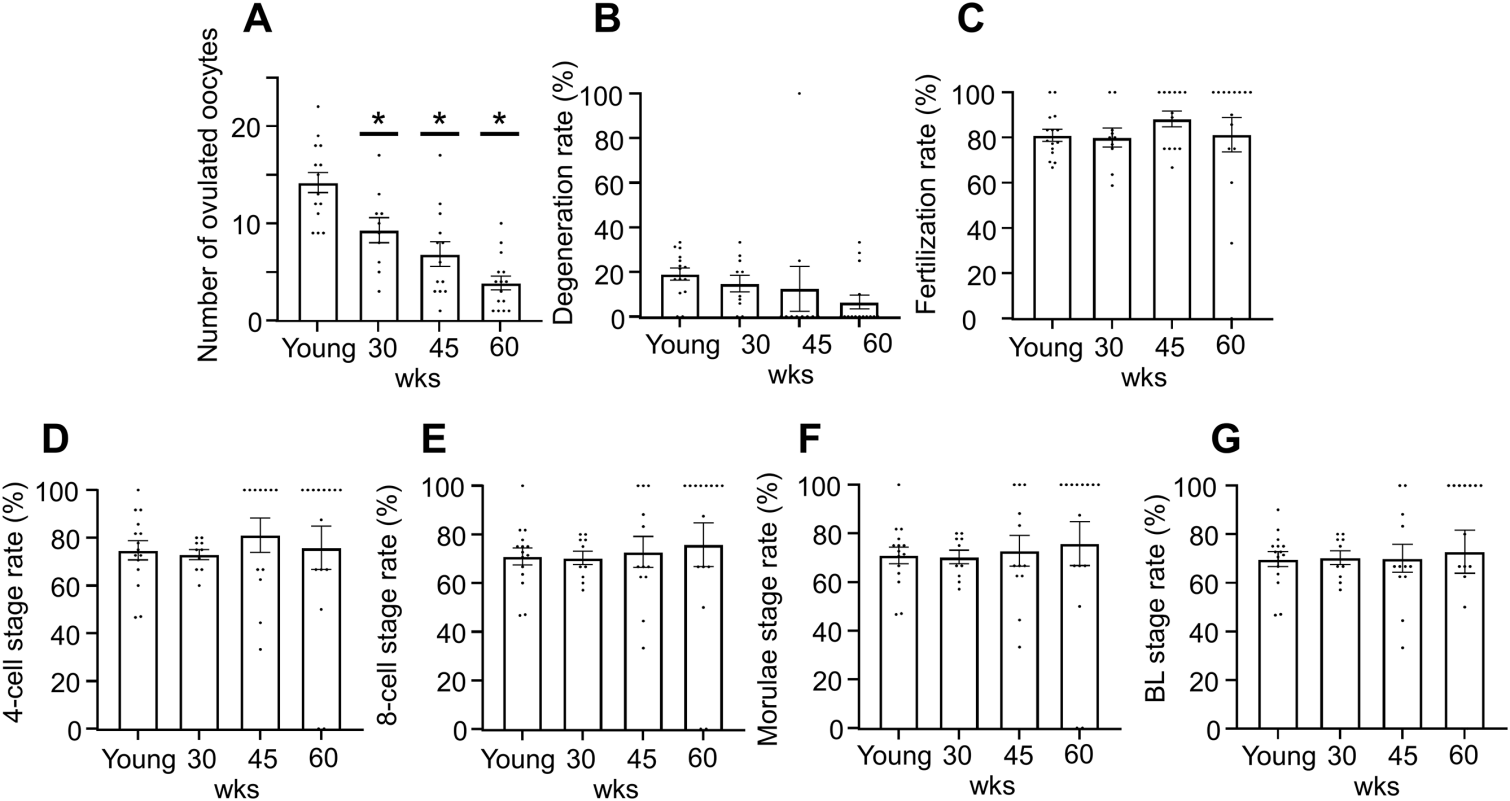
Age-dependent decline in ovulation with preserved fertilization capacity. Mice were treated with hCG to induce ovulation, followed by in vitro fertilization (n = 10–15 / group). This figure shows fertility parameters in young (4–5 weeks of age), 30, 45, and 60 weeks of age mice: (A) Number of ovulated oocytes after hCG injection. (B) Degeneration rate of ovulated oocytes (%). (C) Fertilization rate (2-cell stage/MII oocytes). (D) Embryo development rate from 2-cell stage to 4-cell stage (4 cells/2 cells); (E) Development rate from 2-cell stage to 8-cell stage (8 cells/2 cells); (F) Development rate to morula stage (morula/2 cells); (G) Development rate to blastocyst stage (BL/2 cells) BL: blastocyst. wks: weeks. Data are presented as mean ± SEM and analyzed using one-way ANOVA followed by Tukey#39;s post-hoc test (*p < 0.05 compared to young control).

Following ovarian stimulation and ovulation induction, the number of ovulated oocytes decreased significantly with advancing age: young mice yielded 42.3 ± 3.1 oocytes, whereas 30-, 45-, and 60 weeks of age mice produced 31.2 ± 2.8, 22.4 ± 2.1, and 15.6 ± 1.9 oocytes, respectively (Fig 5A; P < 0.05 for all aging groups vs. young group). However, the proportion of morphologically degenerated oocytes remained stable (8.2–11.4%) across age groups (Fig 5B).

Despite the decline in oocyte yield, fertilization rates were maintained in all age groups, ranging from 78.2% to 82.6% (Fig 5C). Early embryonic development was also unaffected by maternal age, with comparable development rates from the 2-cell to 4-cell stage (85.3–89.1%), 2-cell to 8-cell stage (72.4–78.9%), morula formation (61.2–67.8%), and blastocyst formation (45.6–52.3%) across all age groups (Fig 5D-G).

**Fig 6:**
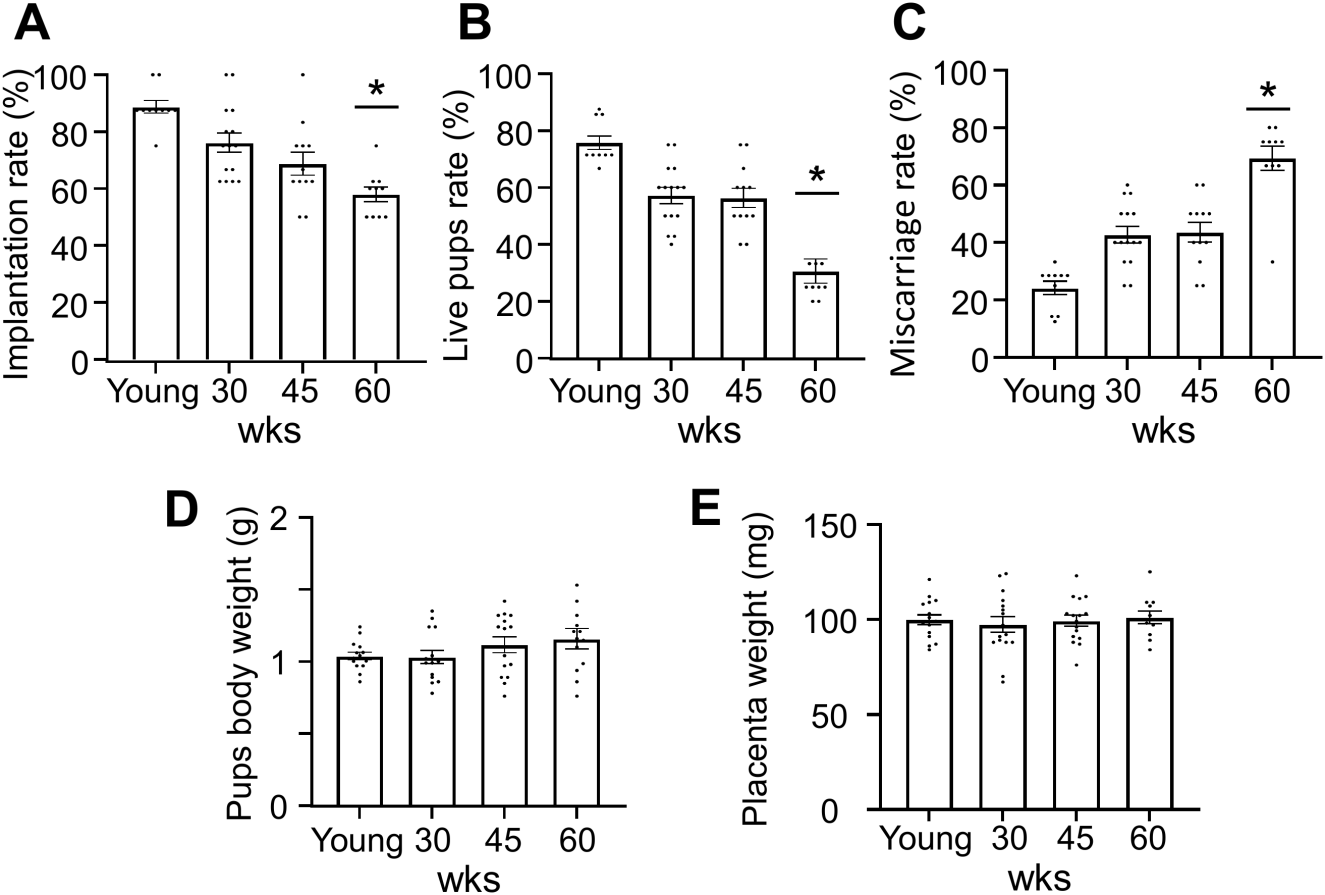
Impaired reproductive outcomes with advancing maternal age. Fertilized eggs obtained from young mice (4–5 weeks old), 30-week-old mice, 45-week-old mice, and 60-week-old mice were transferred as blastocysts into pseudopregnant surrogate mothers (n = 10–15 / group). (A) Implantation rate (implantation site/transferred blastocyst embryo), (B) Live pups rate (live pups/implantation site), (C) Miscarriage rate (miscarriages/implantation site), (D) Pup body weight (g), (E) Placental weight (mg). Data are presented as mean ± SEM and analyzed using one-way ANOVA followed by Tukey#39;s post-hoc test. wks: weeks. *p < 0.05 compared to young control.

In contrast, post-embryo transfer outcomes exhibited significant age-related deterioration. Implantation rates in 60 weeks of age mice were approximately half of those observed in young mice (78.4% to 38.1%, P < 0.05) (Fig 5A). Live pups rates declined from 82.3% in young mice to 43.2% in 60 weeks of age mice (P < 0.01) (Fig 6B), while miscarriage rates increased from 12.1% to 31.8% (P < 0.01) (Fig 5C). Pups body weights (1.42–1.48 g) (Fig 6D) and placenta weights (78.2–84.6 mg) (Fig 6E) remained comparable across all age groups.

## Discussion

This study investigated the relationship between age-related changes in p16 levels in mouse ovaries and the decline of reproductive function in the mouse strain. Our analysis revealed that p16 mRNA and protein levels increased progressively with age in ovarian tissue, and this change was particularly prominent in somatic cells such as granulosa cells and theca cells. In contrast, p16 levels were significantly lower in oocytes and blastocyst-stage embryos. Regarding reproductive function assessment, while ovulation numbers decreased with age, fertilization rates and embryo development rates showed no significant changes. The decline of reproductive performance was pronounced post-implantation. These results suggest that increased p16 levels in ovarian somatic cells may be associated with age-related ovarian dysfunction and reproductive performance deterioration.

Quantitative RT-PCR analysis of age dependent p16 level changes across various organs revealed that cellular senescence progression differs among tissues. Specifically, p16 gene expression increased significantly with age in ovaries, uterus, kidneys, and liver, while no clear changes were observed in brain, heart, and lung. This result is consistent with previous studies reporting organ-specific accumulation patterns of p16-positive cells [3]. The differential aging progression and patterns of p16 accumulation among tissues are influenced by multiple factors [3,11]. Differences in cell types and regenerative capacity among tissues are reported to affect aging progression. Additionally, differences in mitochondrial function, metabolic activity, and oxidative stress tolerance among tissues are reported to influence aging progression rates [16,17]. Other factors, including microenvironmental influences, chronic inflammation, and local hormones and cytokines also affect the aging progression. In ovaries, during follicular development, granulosa cell division is particularly active, making DNA damage and telomere shortening associated with cell division likely to accumulate. Furthermore, inflammatory environments and tissue damage are reported to induce cellular senescence and increase p16 gene expression [18]. The cyclical nature of ovulation creates a unique aging environment not found in other organs [19]. The regular inflammatory responses accompanying ovulatory cycle, along with repeated tissue damage and repair processes, may chronically stimulate cellular senescence pathways [20]. This chronic inflammatory stress and accumulation of cellular damage may contribute to accelerated ovarian aging and earlier increases in p16 levels compared to other organs.

This study demonstrated that p16 levels increased in ovarian tissue in an age-dependent manner. p16 is widely recognized as a biomarker of cellular senescence and increases with age in various tissues. Previous reports have shown increased p16 levels with age in mouse and human ovaries [3,12,21], with significant increases in p16 gene expression specifically reported in the ovarian stroma between 2–4 months and 8–10 months of age in mice [22]. In contrast, four time points from young age to 30, 45, and 60 weeks were compared, observing pronounced age-related increases in p16 levels not only in the stroma but also in corpora lutea and corpora albicantia. The physiological tissue regression of corpora lutea [23] and inflammatory microenvironments in corpora lutea [21] may contribute to increased p16 levels in these regions. Progressive increases in p16 mRNA levels and p16 protein levels from 30 to 60 weeks were also confirmed, supporting previous findings that increased p16 gene expression is involved in ovarian aging processes [3,12,21].

The levels of p16 in ovarian cells varied with age, depending on cell type. A significant increase in p16 levels was observed in somatic cells, including cumulus cells, granulosa cells, and theca cells; however, no significant age-related changes were observed in MII-stage oocytes or blastocyst-stage embryos. Previous reports have shown that single-cell transcriptomic analysis of MII-stage oocytes and cumulus cells revealed that MII-stage oocytes exhibit large-scale gene expression changes in the late reproductive phase (14 months of age), whereas cumulus cells show gradual changes from the early (2 months) to the mid (9 months) phase [24], suggesting that age-related gene expression changes are not synchronized between oocytes and surrounding cumulus cells. Consistent with these results, our data also showed that p16 level changes differ between oocytes and somatic cells, with p16 levels in oocytes being lower than those in cumulus cells. In contrast, an earlier study demonstrated that p16 levels did not change in young cumulus cells compared to aging cumulus cells using transcriptomic analysis [24]. However, our quantitative RT-PCR analysis revealed a significant increase in p16 levels in aging cumulus cells. This discrepancy is primarily attributed to differences in the mouse strain used and the analytical methods employed, namely RNA-seq and quantitative RT-PCR. These differences are likely responsible for the divergent results in detecting p16 levels. A key source of such discrepancy is likely the difference in sensitivity and dynamic range between qRT-PCR and RNA-seq, especially for low-abundance transcripts. In addition, inter-individual biological variability and differences in sample processing may further contribute to variation across studies.

In this study, ICR mouse ovulation numbers decreased significantly with age, but fertilization rates and embryo development competence showed no differences between age groups. These results suggest that while oocyte quantity decreases with age, fertilization rates and blastocyst development capacity are maintained. Previous studies have also reported age-related decreases in ovulation numbers in mice [25,26]. Regarding fertilization rates and embryo development capacity, previous studies using inbred strains have reported age-dependent declines, whereas in the present study using non-inbred strains, age did not affect fertilization rates or embryo development capacity. Previous studies were conducted in inbred mouse strains, while a non‑inbred line was used in the present study. Because fertilization and embryo development rates vary with mouse genotype, and reproductive performance is generally lower in inbred than in outbred females and males, differences in genetic background may underlie the discrepant findings [27,28]. ICR mice are known to exhibit high reproductive performance with remarkable stability across various biological and environmental conditions, compared to inbred strains. This resilience to environmental and biological stresses is attributed to the genetic diversity conferred by their outbred stock status. Such unique properties of ICR mice likely explain why age-related declines in fertilization and embryo development were not observed in our study.

Regarding reproductive outcomes after embryo transfer of embryos from young and aging mice to young recipients, declining trends were observed with age. Sixty-week-old embryos showed significantly decreased implantation and live birth rates, with increased miscarriage rates. These results suggest that aging is associated with impaired post-implantation development of oocytes in mice. Combined with the observation that aging effects were not observed in early embryo development, the effects of aging on mouse oocytes may more strongly influence post-implantation developmental processes than early embryo development.

In the present study, age-related upregulation of p16 was observed in follicular somatic cells, particularly in cumulus, granulosa cells and theca cells, whereas no significant change was detected in oocytes or blastocysts. Cumulus cells play important roles in supporting oocyte maturation and developmental capacity, contributing to oocyte quality through paracrine signaling and metabolic cooperation [29]. Additionally, senescence of somatic cells in follicles surrounding oocytes has been reported as a contributing factor to age-related oocyte quality decline [30]. However, the relationship between gene expression in cumulus cells and oocyte quality remains poorly understood.

p16 induces irreversible cell cycle arrest through inhibition of CDK4/6, preventing Rb phosphorylation and maintaining cells in a permanent growth-arrested state [16]. Senescent cells further contribute to tissue dysfunction by producing pro-inflammatory cytokines and matrix-remodeling enzymes known as the SASP [18], which may create a chronic inflammatory microenvironment within the ovary. Our finding that p16 upregulation is restricted to somatic cells while oocytes remain unaffected suggests that age-related decline in oocyte quality may be driven by paracrine effects from surrounding senescent somatic cells rather than intrinsic oocyte senescence. This hypothesis is supported by the recent demonstration that exposure of aged oocytes to a young follicular microenvironment restores their developmental competence [30], and raises the possibility that senolytic interventions targeting p16-positive somatic cells could improve ovarian function.

In conclusion, this study links age-related p16 upregulation in ovarian somatic cells to impaired post-implantation development, despite preserved fertilization and preimplantation embryo development. These observations highlight somatic cell senescence as a key factor in ovarian aging and a potential target for future interventions. In line with this concept, a recent study demonstrated that exposure of aged oocytes to a young follicular microenvironment can improve their competence and developmental potential [30]. Together with our observation that age-related upregulation of p16 occurs predominantly in ovarian somatic cells and is accompanied by impaired post-implantation outcomes, these findings further support a close link between ovarian somatic cell aging and reproductive decline. Future research should clarify how ovarian somatic cell senescence affects oocyte quality and evaluate p16 levels in cumulus cells as a marker of oocyte competence, as well as the efficacy of senolytic approaches that eliminate p16-positive senescent cells to improve ovarian function. Although this study demonstrates associations between p16 levels and reproductive outcomes, additional studies, including selective manipulation of p16-positive cells and validation in other models, will be required to establish p16 as a definitive clinical biomarker of ovarian aging.

## Supporting information

S1 Fig mRNA levels of key oocyte-related genes across different cell types. mRNA levels of *Gdf9* (oocyte marker), *Has2* (cumulus cell marker), *Fshr* (granulosa cell marker), and *Cyp17a1* (theca cell marker) was quantified by qRT-PCR in oocytes (OC), cumulus cells (CC), granulosa cells (GC), and theca cells (TC), and normalized to β-actin. Data are presented as mean ± SEM (n = 6–8 per group).
(TIF)

S2 Fig Schematic overview of the in vitro fertilization and embryo transfer procedures. Female ICR mice were administered 15 IU hCG intraperitoneally for ovulation induction without PMSG priming. Cumulus-oocyte complexes were collected from oviducts 14–15 hours after hCG injection (oocyte collection), followed by sperm insemination and co-culture for 6 hours. Fertilization was confirmed by the presence of zygotes, and embryos were cultured from the 2-cell stage for 96 hours until the blastocyst stage. For embryo transfer, blastocyst-stage embryos were transferred into the uteri of pseudopregnant surrogate mothers, and reproductive outcomes were evaluated by Cesarean section on day 15 post-embryo transfer (E18.5).
(TIF)

S1 Table Primer sequences used for qPCR analysis. Forward and reverse primer sequences (5′ to 3′) are listed for *p16*, *Gapdh*, *Gdf9*, *Has2*, *Fshr*, *Cyp17a1*, and *β-actin*.
(TIF)
