# Supplementary figures and images for: Age-related upregulation of p16 expression in mouse ovarian somatic cells correlated with reproductive function decline p16 expression and ovarian aging in mice
Source: PLoS One. 2026 May 8;21(5):e0348870. doi: 10.1371/journal.pone.0348870 (PMC13155578; doi:10.1371/journal.pone.0348870)

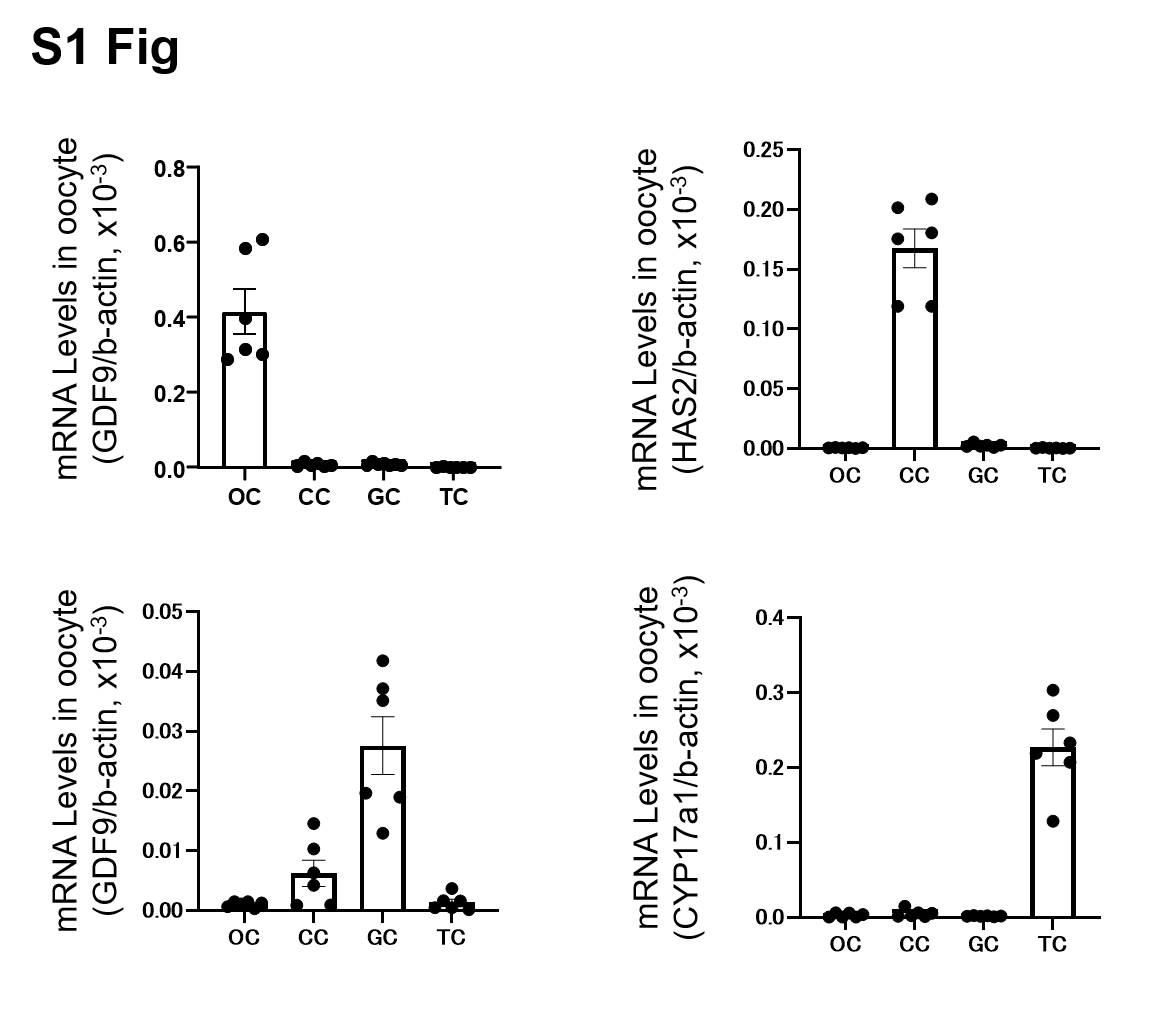

Supplement: S1 Fig — mRNA levels of Gdf9 (oocyte marker), Has2 (cumulus cell marker), Fshr (granulosa cell marker), and Cyp17a1 (theca cell marker) was quantified by qRT-PCR in oocytes (OC), cumulus cells (CC), granulosa cells (GC), and theca cells (TC), and normalized to β-actin. Data are presented as mean ± SEM (n = 6–8 per group). (TIF) [file pone.0348870.s001.tif]

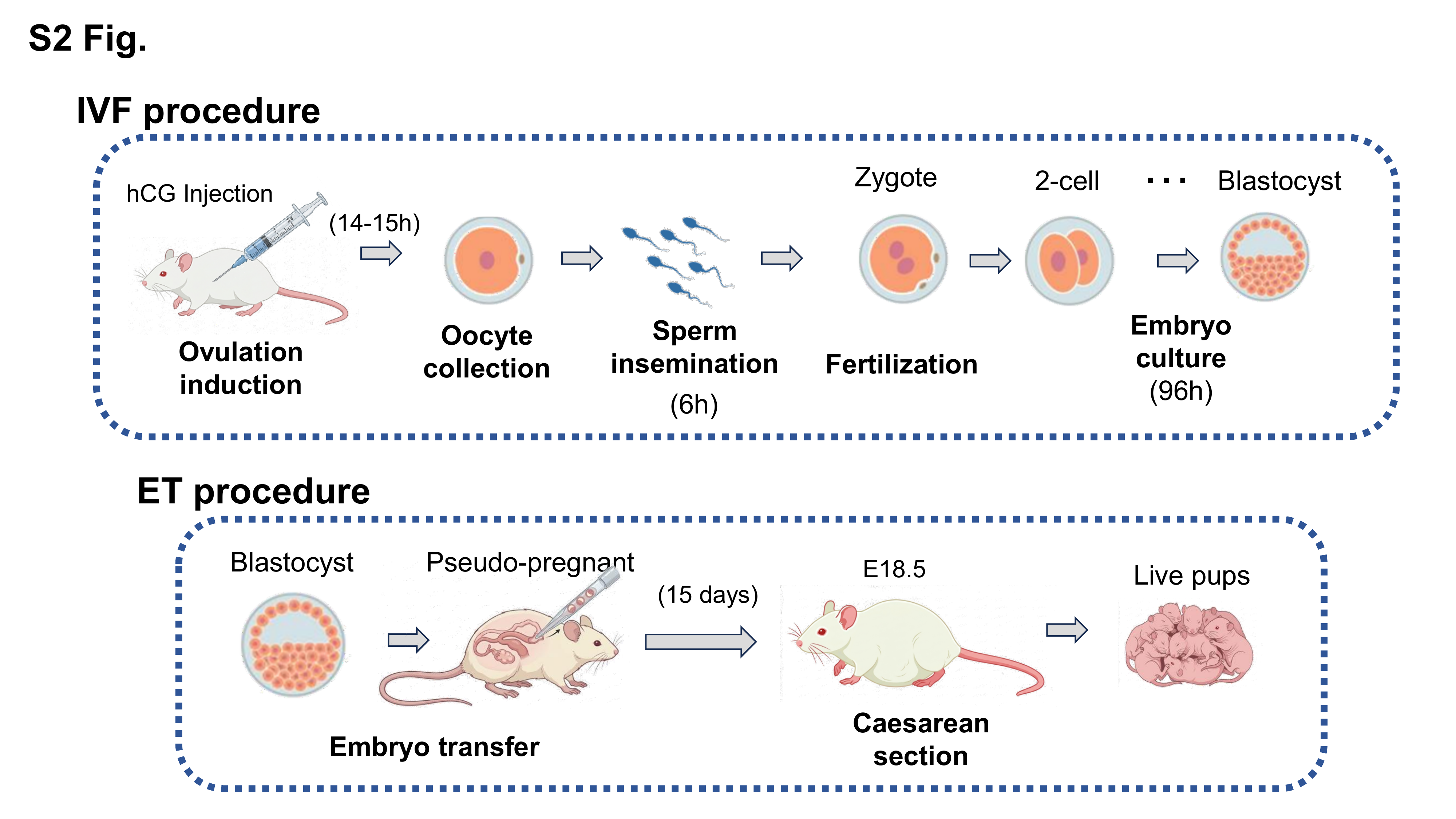

Supplement: S2 Fig — Female ICR mice were administered 15 IU hCG intraperitoneally for ovulation induction without PMSG priming. Cumulus-oocyte complexes were collected from oviducts 14–15 hours after hCG injection (oocyte collection), followed by sperm insemination and co-culture for 6 hours. Fertilization was confirmed by the presence of zygotes, and embryos were cultured from the 2-cell stage for 96 hours until the blastocyst stage. For embryo transfer, blastocyst-stage embryos were transferred into the uteri of pseudopregnant surrogate mothers, and reproductive outcomes were evaluated by Cesarean section on day 15 post-embryo transfer (E18.5). (TIF) [file pone.0348870.s002.tif]

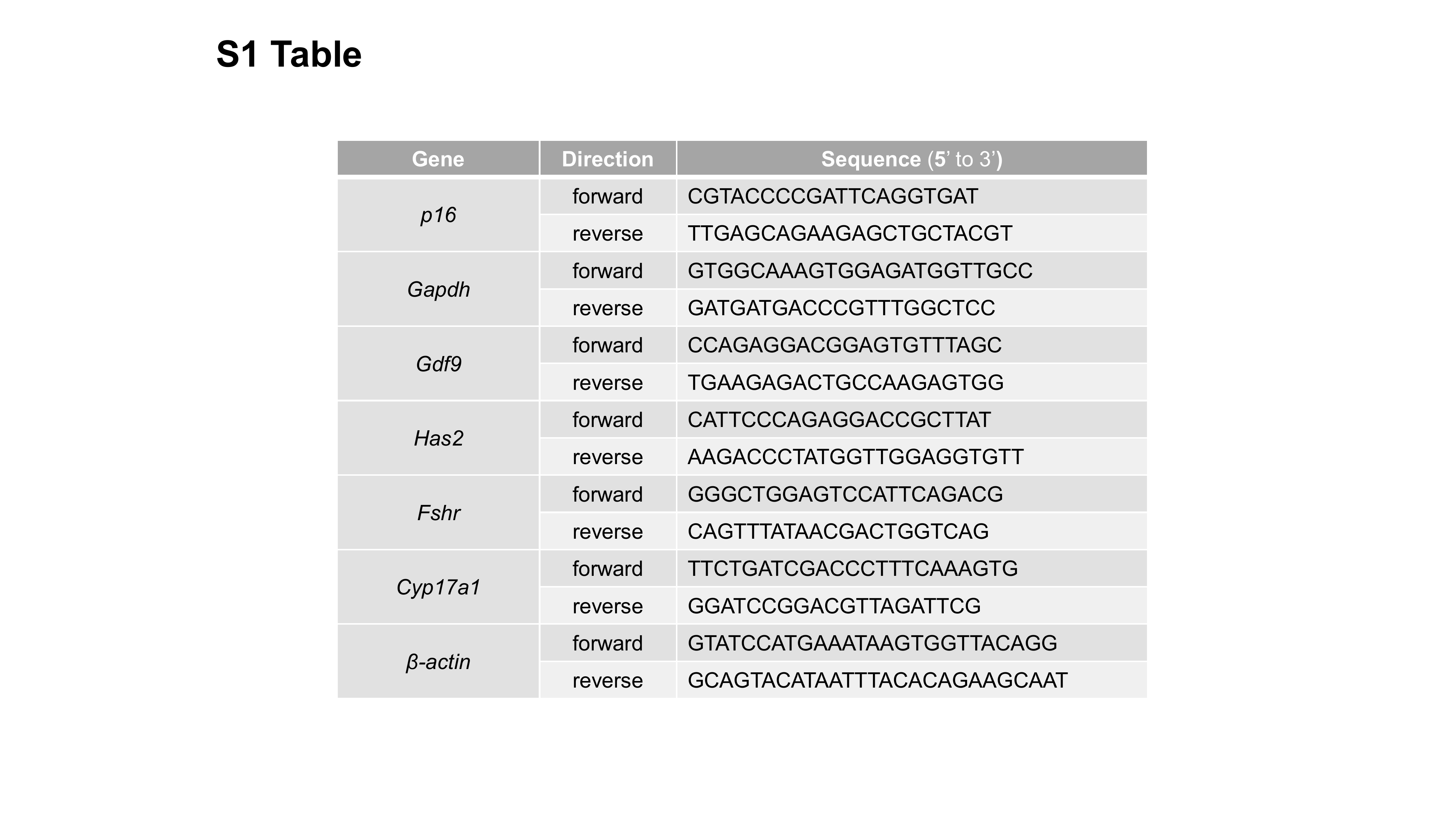

Supplement: S1 Table — Forward and reverse primer sequences (5′ to 3′) are listed for p16, Gapdh, Gdf9, Has2, Fshr, Cyp17a1, and β-actin. (TIF) [file pone.0348870.s003.tif]
